# Laminin-521 Promotes Rat Bone Marrow Mesenchymal Stem Cell Sheet Formation on Light-Induced Cell Sheet Technology

**DOI:** 10.1155/2017/9474573

**Published:** 2017-01-09

**Authors:** Zhiwei Jiang, Yue Xi, Kaichen Lai, Ying Wang, Huiming Wang, Guoli Yang

**Affiliations:** ^1^Department of Implantology, Stomatology Hospital, School of Medicine, Zhejiang University, Yan'an Road, Hangzhou, China; ^2^Department of Oral Medicine, Stomatology Hospital, School of Medicine, Zhejiang University, Yan'an Road, Hangzhou, China; ^3^Department of Oral and Maxillofacial Surgery, Stomatology Hospital, School of Medicine, Zhejiang University, Yan'an Road, Hangzhou, China

## Abstract

Rat bone marrow mesenchymal stem cell sheets (rBMSC sheets) are attractive for cell-based tissue engineering. However, methods of culturing rBMSC sheets are critically limited. In order to obtain intact rBMSC sheets, a light-induced cell sheet method was used in this study. TiO_2_ nanodot films were coated with (TL) or without (TN) laminin-521. We investigated the effects of laminin-521 on rBMSCs during cell sheet culturing. The fabricated rBMSC sheets were subsequently assessed to study cell sheet viability, reattachment ability, cell sheet thickness, collagen type I deposition, and multilineage potential. The results showed that laminin-521 could promote the formation of rBMSC sheets with good viability under hyperconfluent conditions. Cell sheet thickness increased from an initial 26.7 ± 1.5 *μ*m (day 5) up to 47.7 ± 3.0 *μ*m (day 10). Moreover, rBMSC sheets maintained their potential of osteogenic, adipogenic, and chondrogenic differentiation. This study provides a new strategy to obtain rBMSC sheets using light-induced cell sheet technology.

## 1. Introduction

Cell suspensions combined with bioscaffolds are widely used in traditional tissue engineering. However, inflammatory responses are induced during the degradation of bioscaffolds, thus affecting experimental results. More recent attention has focused on cell sheet engineering, whereby cultured cells are harvested as intact sheets along with their deposited extracellular matrix (ECM) and cellular connections, without using any scaffolds. In contrast to single cell suspensions, cell sheets can attach well on host sites with minimal cell loss. Additionally, because the ECM provides a scaffold-like surface, cell sheets can be directly layered to regenerate three-dimensional (3D) tissue-like structures. Cell sheet technology therefore provides several advantages over traditional tissue engineering. A large and growing body of literature has investigated the possibility of applying cell sheets to cardiac regeneration [1], cornea [2], esophagus [3], periodontal tissue [4], and dental implant [5]. Thus far, cell sheet technology is an area of research that is of great interest for tissue engineering and regenerative medicine. This study will report a novel method for the culture of rat bone marrow mesenchymal stem cell sheets, using light-induced dissociation from the culture substrate. Despite the numerous studies that explore the methods of construction of rBMSC sheets, we have not found a study that has developed rBMSC sheets with light-induced cell sheet technology.

Current methods for cell sheet harvesting include temperature- [6], electricity- [7], magnetism- [8], and pH change-induced methods [9]. To date, most of these methods have taken advantage of surface property variations to induce cell detachment. In a previous study [10], we developed light-induced cell sheet technology as a simple, rapid, and effective method based on altering the surface wettability. Cell sheets can detach on a TiO_2_ nanodot-coated quartz substrate after UV365 illumination. Moreover, TiO_2_ nanodots and UV365 illumination are safe for cell sheets. However, only a few cell types of cell sheets (MC3T3 [11] and NIH3T3 [12]) have been developed with this technology. In this study, rBMCSs were chosen to develop cell sheets using light-induced cell sheet technology.

Murine BMSCs are a promising source of cells for cell transplantations owing to their multilineage differentiation potential [13]. In contrast to the terminally differentiated cells, murine BMSCs are immune-tolerated following allogeneic stem cell transplantation [14]. In addition, rBMSCs are quite easy to obtain and proliferate well in vitro. Moreover, rBMSC sheets are beneficial for various tissue engineering applications. It has been reported that rBMSC sheets can be used in enhancing osseointegration [5], bone defect regeneration [15], and treatment of bone fractures [16]. Until now, rBMSC sheets have been developed on untreated dishes [17] and temperature-responsive dishes [18]. However, the methods of culturing rBMSC sheets are critically limited. Light-induced cell sheet technology can potentially provide a more convenient and rapid approach for cell sheet engineering. Unfortunately, our preliminary experiments determined that rBMSCs were difficult to form cell sheets on the TiO_2_ nanodot films.

In order to obtain intact rBMSC sheets and maintain their multipotency, we tried to coat adhesion molecules on the TiO_2_ nanodot films. In the preliminary experiment of measuring the effects of rat fibronectin, human recombinant laminin-521, laminin-511, laminin-421, and laminin-111 on the formation of cell sheets, human recombinant laminin-521 was found to have a higher rate of success. Moreover, human recombinant laminin-521 is readily-available and lower cost than other human laminin isoforms and rat fibronectin. Recently, laminin-521 has been reported to promote human stem cells adhesion and maintain their pluripotency [19]. Laminin-521 (a5*β*2*γ*1) is expressed early in embryonic development [20] and is shown to support embryonic cells growth in monolayer plate cultures [21]. Laminin-521 exerts effects on proliferation, migration, phenotype stability, and survival of various cell types. Cultures of human embryonic stem cells, human induced pluripotent stem cells, and human mesenchymal stem cells can be supported by laminin-521 [21–23]. However, although laminin-521 was developed for culturing of human stem cells, no study reported whether it had effects on rBMSCs. The aim of this study is to investigate the feasibility of fabricating an intact rBMSC sheet through laminin-521 by light-induced cell sheet technology.

## 2. Materials and Methods

### 2.1. Preparation of TiO_2_ Nanodot Films and Immobilization of Laminin-521

TiO_2_ nanodot films were prepared on a quartz glass substrate through phase separation-induced self-assembly [10]. A precursor sol containing acetylacetone (AcAc, Lingfeng Chemical Reagent, AR, >99%), titanium tetrabutoxide (TBOT, Sinopharm Chemical Reagent, CP, >98%), and polyvinyl pyrrolidone (PVP, K30, Sinopharm Chemical Reagent, AR, >99%) was spin-coated on the quartz glass substrates at 8000 rpm for 40 s and further heated at 500°C. Laminin-521 (Biolamina, Sundbyberg, Sweden, 100 *μ*g/mL) was dissolved in DPBS with calcium and magnesium to obtain 1200 ng/mL solution. TiO_2_ nanodot films were immersed in the solution overnight at 4°C in a sterile environment.

### 2.2. Isolation and Culture of Rat BMSCs

The Institutional Animal Care and Use Committee of Zhejiang University, Hangzhou, China, approved the animal experiments in this study. Three-week-old male Sprague-Dawley rats were used. Isolation and culture of rBMSCs were performed as described elsewhere [24]. Briefly, rBMSCs were aspirated from the bone marrow, and the obtained cells were cultured in alpha-modified minimum essential media (aMEM, Gibco, USA) containing 10% fetal bovine serum (FBS; Gibco, USA), 0.272 g/L L-glutamine (Sigma, USA), and 1% antibiotic solution (penicillin and streptomycin (Gibco, USA)). The rBMSCs were cultured for three passages and then used in follow-up experiments.

### 2.3. Cell Adhesion Assay

TiO_2_ nanodot films were coated with increasing concentrations of laminin-521 (0, 300, 600, 900, 1200, 1500, 2000, and 3000 ng/mL) at 4°C overnight (15 hours). In a 24-well plate, rBMSCs were seeded on 1 × 1 cm^2^ TiO_2_ nanodot films at a density of 3 × 10^4^ cells/well. After incubating the cells for 0.5 h and 2 h, the medium was removed and washed three times with 1x PBS. The cells were incubated in medium supplemented with 10% Alamar Blue dye (Invitrogen, Carlsbad, CA) for 4 h at room temperature. An aliquot (100 *µ*L) of medium from each sample was read at 540/590 nm in a microplate reader (Turner Biosystems, Sunnyvale, CA). Medium supplemented with 10% Alamar Blue dye was used as a negative control.

Cells were seeded on nanodot films coated without or with 1.2 *μ*g/mL laminin-521. After 2 h and 4 h, rBMSCs were fixed in 4% paraformaldehyde for 30 min. Fluorescein isothiocyanate-conjugated phalloidin (FITC-phalloidin, Buchs, Switzerland) was used to label actin filament (F-actin). As a counterstain for nuclei, 4′-6 diamidino-2-phenolindole (DAPI, Sigma, USA) was used. Cells were incubated with FITC-phalloidin for 60 min and washed three times with 1x PBS. Then they were incubated with DAPI for 5 min and washed three times with 1x PBS. The cell morphology and cytoskeletal arrangement were analyzed by fluorescence inversion microscope system (AX10, Zeiss, Germany). Cell morphology was described using a shape factor *ϕ*, which was expressed as *ϕ* = 4*πA*/*p*^2^ (*A* was the footprint area and *p* was the perimeter of the cell) [25]. The better the cells adhered on the substrates, the smaller the value of *ϕ* was.

### 2.4. Protein Adsorption

TiO_2_ nanodot films were coated with 1.2 *μ*g/mL laminin-521 at 4°C overnight. Films immersed in DPBS were used as a negative control. Micro BCA protein assay (Pierce Chemical Co, Rockford, IL) was conducted to analyze protein adsorption. After adsorption, nonadherent solutions were removed. An aliquot (1 mL) of the initial and removed solution were mixed with 1 mL of micro bicinchoninic acid (BCA) working reagent in a test tube and incubated at 60°C for 60 min and then measured using a multifunctional microplate reader (SpectraMax M5, MD, USA) at 592 nm. The concentration of adsorbed proteins was determined by subtracting the residual proteins from the initial added proteins.

### 2.5. Cell Sheet Formation and Detachment Assay

Nanodot films were coated with 1.2 *μ*g/mL laminin-521 at 4°C overnight. In a 24-well plate, rBMSCs were seeded on the nanodot films at a density of 3 × 10^4^ cells/cm^2^. Nanodot films without coating were used as a negative control. When the cells were confluent after 2-3 days in culture, the medium was replaced every day. At 1 day and 3 and 5 days after seeding, rBMSCs were observed by a phase-contrast microscope (CKX41, Olympus, Japan). Cells formed a monolayer after being cultured for 5 days and then were illuminated under 5.5 mW/cm^2^ power of UV365 for 30 min to obtain cell sheets.

### 2.6. Proliferation, 8-Hydroxy-Deoxyguanosine, and Live-Dead Staining Assay of Cell Sheets

After UV illumination, the harvested cell sheets were digested by trypsin. The control group was cell sheets which were digested by trypsin directly without UV illumination. Then cells were reseeded on a 24-well plate. The viability of rBMSCs was determined using the Alamar Blue colorimetric assay at 1 day, 3, 5, and 7 days, in a similar procedure as mentioned above.

A rat 8-hydroxy-deoxyguanosine (8-OH-dG) ELISA kit (Cusabio, China) was used to evaluate the integrity of DNA. A final cell density of 3 × 10^4^ cells/cm^2^ BMSCs was seeded on laminin-521-coated TiO_2_ nanodot films and cultured for 5 days to form cell sheets. After adding 500 *μ*L serum-free medium to each well and 30 min of UV365 illumination, the culture medium was collected. The relative amounts of 8-OH-dG in the collected culture medium were quantified following manufacturer's instructions.

The viability of cell sheets was further evaluated using a live-dead staining method, based on a simultaneous determination of live and dead cells with Calcein-AM and PI. Dead cells would fluoresce red by PI, while live cells would fluoresce green by Calcein-AM. The detached cell sheets were rinsed three times with 1x PBS. Cell sheets were incubated with appropriate Calcein-AM for 5 min at room temperature in the dark. After the sheets were rinsed three times with 1x PBS, PI (1 : 200 in 1x PBS) was added and incubated for 20 min at room temperature in the dark. Then the sheets were rinsed another three times with 1x PBS. Cell sheets were observed using a fluorescence inversion microscope (IX81, Olympus, Japan). As a negative control, a piece of cell sheet was treated in 30% ethanol for 30 min, and the same procedure was repeated.

### 2.7. Reattachment, Collagen Type I Immunofluorescence, and Scanning Electron Microscopy (SEM) Analysis of Cell Sheets

The obtained cell sheets were plated on 24-well plates to evaluate their reattachment ability at 12 hours, 1 day, and 3 and 5 days observed by phase-contrast microscope. Furthermore, cell sheets were plated on titanium disks, which were often used in dental implant research studies [26]. At day 7, cytoskeleton and nuclei of cell sheets were stained with FITC-phalloidin and DAPI.

The detached cell sheets were first fixed in 4% paraformaldehyde for 30 min. Cells were incubated with an optimal concentration of anti-collagen type I antibody (1 : 200, Abcam, ab90395) overnight at 4°C and then incubated with anti-mouse Alexa Fluor 488 secondary antibody (1 : 500, Abcam, ab150117), followed by rinsing three times with PBS. Nuclei were counterstained with DAPI.

After detachment, rBMSC sheets were immediately fixed with 2.5% glutaraldehyde at 4°C overnight. The negative control group was cells reseeded on TiO_2_ nanodot films for 30 min after cell sheets were digested by trypsin directly without UV illumination. The fixed cell sheets and cells underwent dehydration in a graded series of ethanol solutions; then they were immersed in HMDS for 10 min and air-dried before observation by a SEM (SU-8010, Hitachi, Japan).

### 2.8. Hematoxylin and Eosin (H&E) Staining and Measurement of Cell Sheet Thickness

At day 5, fabricated BMSC sheets were fixed by 4% paraformaldehyde and underwent dehydration, embedding, paraffin sectioning, and processing for H&E staining. The structure of the cell sheet was then observed by microscope. After culturing for 5, 7, and 10 days, cell sheets were harvested and fixed by 4% paraformaldehyde for 30 min. FITC-phalloidin dye was used to stain the F-actin of the cell sheet while nuclei were labeled with DAPI. The dye was removed and washed with 1x PBS before observation with confocal microscopy (Nikon A1 Ti, Tokyo, Japan). To determine the thickness of the cell sheet, the* z*-direction slicing mode was used and an average of nine points was taken.

### 2.9. Differentiation Assay

#### 2.9.1. Osteogenic Differentiation

After fabricating the BMSC sheets, they were reattached in a 24-well plate. The nutrient solution was replaced with osteogenesis-inducing liquid (Cyagen Biosciences Inc., Guangzhou, China) containing 10% FBS, 100 U/mL penicillin-streptomycin, 0.2 mM ascorbate, 10 mM *β*-glycerophosphate, and 10^−7^ M dexamethasone. For the control group, cell sheet nutrient solution (which included 10% FBS concentration of *α*MEM) was used. Cells were incubated at 37°C in 5% CO_2_ and culture medium was changed every 3 days. After 21 days, alizarin-red staining was then applied following the manufacturer's protocol. Two groups were rinsed with 1x PBS three times, and calcium-nodule formation was observed under phase-contrast microscopy.

#### 2.9.2. Adipogenic Differentiation

Culture medium was removed and replaced with adipogenic induction medium (Cyagen Biosciences Inc., Guangzhou, China). Adipogenic differentiation was stimulated using three cycles of induction medium A (stimulatory supplements including dexamethasone, 3-isobutyl-1-methylxanthine, insulin, and indometacin) for 3 days and maintenance medium B (stimulatory supplements including insulin) for 24 hours. Control group wells were cultured in cell sheet nutrient solution. Cells were incubated at 37°C in 5% CO_2_ over a period of 3 weeks. At harvest, the medium was gently aspirated, and then cells were fixed in 4% paraformaldehyde for 30 min and stained with Oil Red O following the manufacturer's protocol. Red lipid droplets were observed under the phase-contrast microscope.

#### 2.9.3. Chondrogenic Differentiation

According to the manufacturer's instructions (Cyagen Biosciences Inc., Guangzhou, China), a standard pellet culture was performed. The culture medium which contained 0.1 *μ*L mL^−1^ dexamethasone, 3 *μ*L mL^−1^ ascorbate, 10 *μ*L mL^−1^ ITS+Supplement, 1 *μ*L mL^−1^ sodium pyruvate, 1 *μ*L mL^−1^ proline, and 10 *μ*L mL^−1^ TGF-*β*3 was changed every three days. After four weeks of chondrogenic incubation, the pellet was fixed in 4% paraformaldehyde, embedded in optimum cutting temperature compound (OCT), and cut into slices. Then the slices were stained with Alcian Blue for 30 min, washed three times with 1x PBS, and observed under the phase-contrast microscope.

#### 2.9.4. RNA Extraction and Real-Time Reverse Transcriptase-Polymerase Chain Reaction (RT-PCR) Analysis

The fabricated rBMSC sheets were reattached onto a 6-well plate with induction of osteogenic, adipogenic, or chondrogenic medium for 7 days. The control group was cultured in cell sheet nutrient medium. Total RNA from each group was extracted using a TRIzol reagent (Invitrogen) and then cDNA was generated by reverse transcription using a 20 *µ*L reaction mixture containing Superscript III RNase H-reverse transcriptase (Invitrogen). SYBR Green PCR assays were performed using these cDNA samples on a MX3000P Real-Time qPCR System (Stratagene, CA, USA). The sequences of the PCR primers for alkaline phosphatase* (ALP)*,* col1a1, runx2, PPARγ, AP2, leptin (LEP)*,* SOX9*, aggrecan* (ACAN)*,* col2a1,* and*β-actin *are presented in Table 1 (TAKARA, Dalian, China). The thermal cycling protocol was performed as follows: denaturation at 95°C for 3 min and 40 cycles of amplification and quantification (95°C for 120 s, 62°C for 40 s). Fluorescence data was analyzed to obtain CT values at the end of each run. The 2^−ΔΔCT^ method was used to calculate gene expressions, while CT values from samples were averaged and calibrated in relation to *β*-actin CT values.

### 2.10. Statistical Analysis

All data were expressed as mean ± standard deviation. They were analyzed using the SPSS 17.0 software package by unpaired Student's* t-*test. Differences were considered statistically significant at *P* < 0.05.

## 3. Results

### 3.1. Cell Adhesion Assay and Protein Adsorption

**Figure 1:**
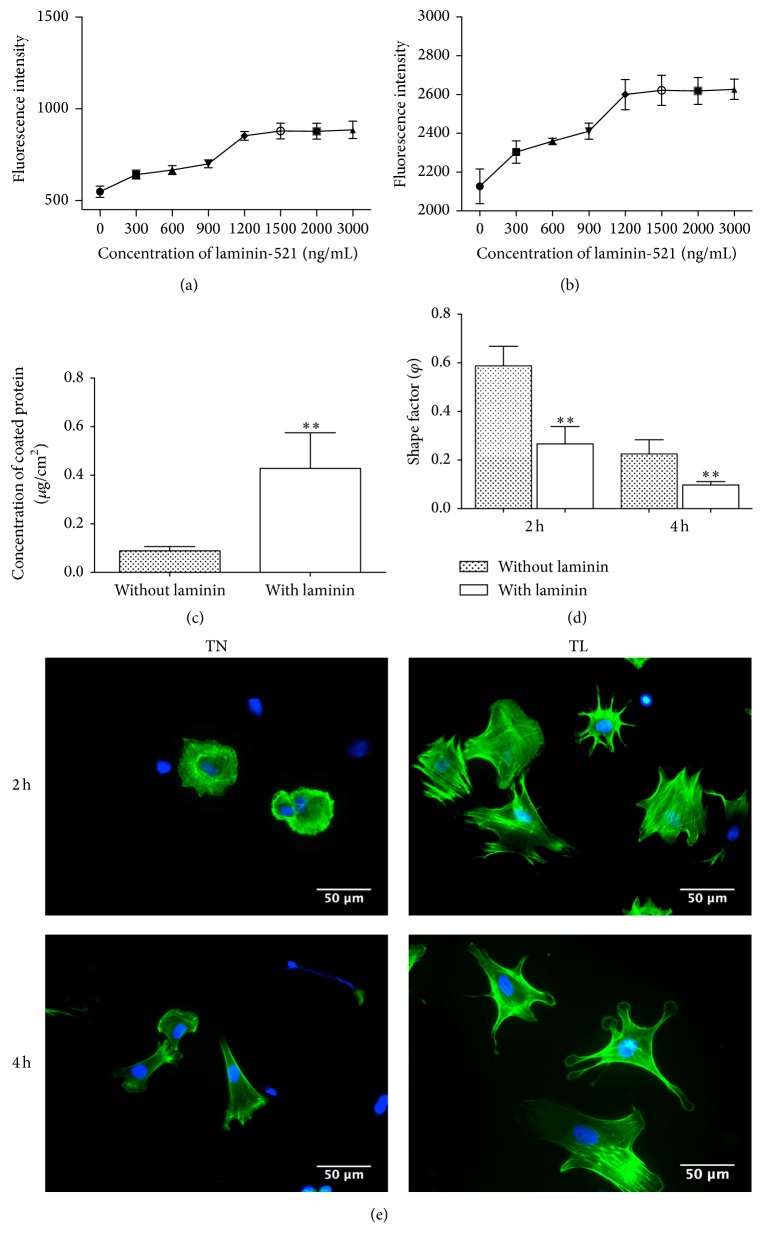
Adhesion assay of rBMSCs on nanodot films coated with (TL) or without (TN) laminin-521. Attached rBMSCs on different concentration of laminin-521-coated samples after incubation for 0.5 h (a) and 2 h (b) tested by Alamar Blue. Concentration of coated laminin-521 measured by micro BCA assay (c). Ability of adhesion measured by cytoskeleton (green) and nuclei (blue) double-labeled method after culturing on TN and TL samples for 2 h and 4 h (d), (e). Scale bar: 50 *μ*m. ^*∗∗*^*P* < 0.01.

**Figure 2:**
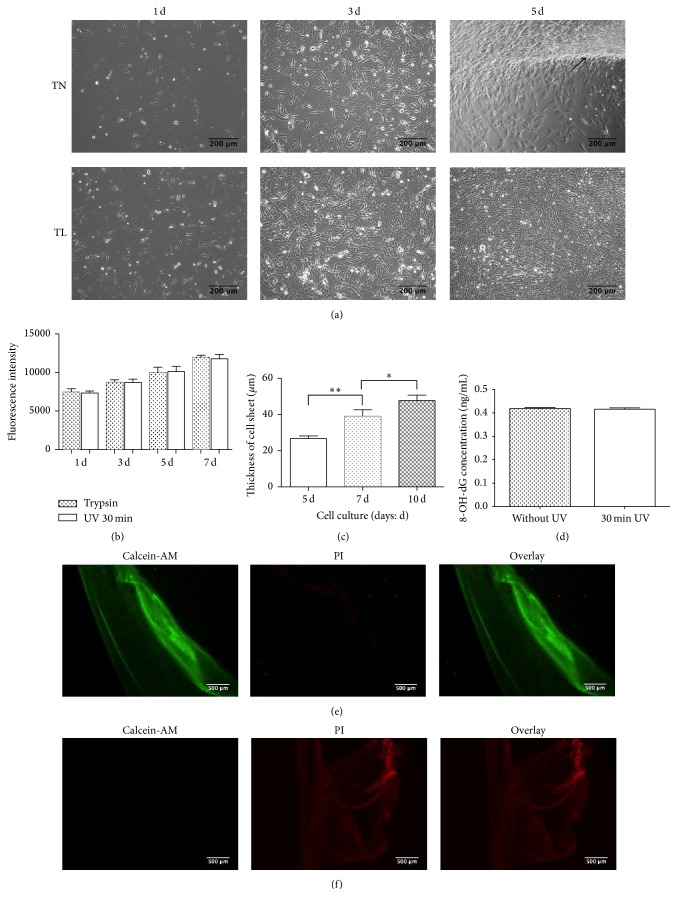
Morphology of rBMSCs cultured on TN or TL samples after seeding for intended time observed by phase-contrast microscope (a). Proliferation assay of rBMSC sheets treated with UV illumination or trypsin tested by Alamar Blue (b). Thickness of fabricated cell sheets at day 5, day 7, and day 10 observed by laser confocal microscopy (c). Oxidative damage of DNA assessed using 8-OH-dG assay (d). Live-dead staining of rBMSC sheets after illumination of UV365 for 30 min (e). rBMSC sheets with the treatment of 30% ethanol were stained as negative control group (f). ^*∗*^*P* < 0.05, ^*∗∗*^*P* < 0.01. Scale bar: (a): 200 *μ*m; (e), (f): 500 *μ*m.

To evaluate the modulatory effect of laminin-521 on rBMSCs attachment, the cells were cultured on nanodot films coated with (TL) or without (TN) laminin-521. The fluorescence intensity revealed that more rBMSCs were attached on TL samples than TN samples after incubation for 0.5 h (Figure 1(a)) and 2 h (Figure 1(b)) (*P* < 0.05). More attached cells were also detected on 300 ng/mL laminin-521-coated nanodot films than the negative control group. When the concentration of laminin-521 was further increased, the number of BMSCs increased until the concentration was over 1200 ng/mL (*P* < 0.01). To further evaluate the interaction of rBMSCs with laminin-521 during the early stage of adhesion, rBMSCs were seeded on either 1200 ng/mL laminin-521-coated nanodot films or noncoated films for 2 h and 4 h to examine the F-actin distribution and cell spreading morphology.  Figure 1(e) showed that cells on TL samples exhibited more fibrillar adhesions in the cytoplasm and adhered faster than those incubated on TN samples. The shape factor *ϕ* of cells on TL samples was lower than that on TN samples (*P* < 0.01) (Figure 1(d)). The observation implied that laminin-521 improved cell spreading and adhesion. To measure the quantity of laminin-521 on TiO_2_ nanodot films, micro BCA protein assay was conducted. The results showed that films were coated with 0.43 ± 0.1 *μ*g/cm^2^ laminin-521 when the initial concentration of laminin-521 was 1.2 *μ*g/mL (Figure 1(c)).

### 3.2. Cell Sheet Formation and Detachment

To evaluate the potential of laminin-521 in cell sheet formation application, the rBMSCs were seeded on 1.2 *μ*g/mL laminin-521-coated nanodot films and TN samples for 1, 3, and 5 days. As shown in Figure 2(a), rBMSC sheets could hardly form on TN samples. Cells partially detached from TN samples at day 5 and failed to form cell sheets. Conversely, rBMSCs attached well on TL samples and formed intact cell sheets after culturing for 5 days. Cell sheets were obtained with illumination of UV365 for 30 min. The result revealed that laminin-521 was beneficial for rBMSC sheet formation. Based on the above observations, all subsequent experiments were conducted using 1.2 *μ*g/mL laminin-521-coated nanodot films.

### 3.3. Proliferation, 8-OH-dG, and Live-Dead Staining Assay of Cell Sheets

To further determine the effects of UV365 and laminin-521 on fabricated cell sheets, proliferation, 8-OH-dG, and live-dead staining assay were conducted. After cell sheets were digested by trypsin before or after UV illumination, cells were reseeded on 24-well plate. The results showed that there was no significant difference in cell proliferation between trypsin and UV-treated cell sheets (Figure 2(b)). To evaluate the DNA integrity of the obtained cell sheets, 8-OH-dG assay was conducted. As shown in Figure 2(d), there was no significant difference between the UV365 illumination group and the control group in 8-OH-dG concentration. This result showed that rBMSC sheets obtained with laminin-521 and UV365 illumination system did not have any significant DNA oxidative damage. Moreover, the survival of rBMSC sheets was examined immediately by live-dead staining. As shown in Figure 2(e), live cells were stained with Calcein-AM (green), while dead cells were red (PI). In the negative control, cell sheets immersed 30% ethanol treated for 30 min, and all the cells were dead and stained with red (Figure 2(f)). Illuminated rBMSC sheets had good viability as there were only a few of cells stained with red (Figure 2(e)). These results implied that rBMSC sheets harvested by light-induced detachment method on laminin-521 coated samples were viable.

### 3.4. Reattachment, Collagen Type I Immunofluorescence, and SEM of rBMSC Sheet

**Figure 3:**
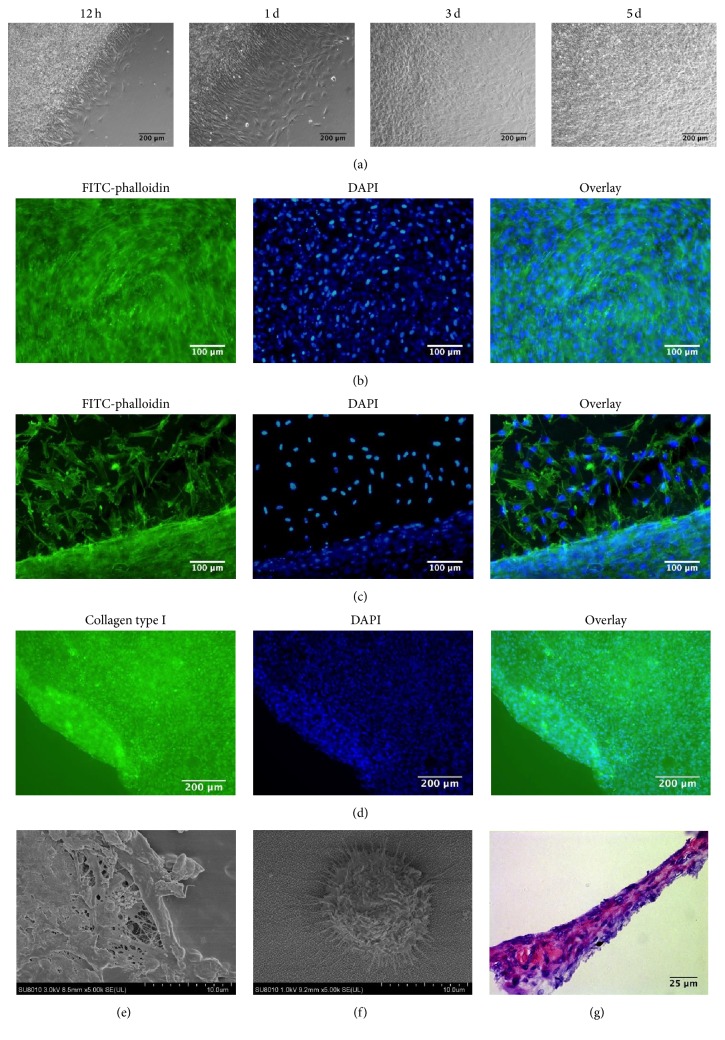
Reattachment of rBMSC sheets on 24-well plate (a) observed at 12 h, day 1, day 3, and day 5 by phase-contrast microscope. Cytoskeleton and nuclei double-labeled assay and rBMSC sheet observed by fluorescent microscopy in center (b) and at edge (c). Collagen type I immunofluorescence of rBMSC sheet (d). Morphology of detached rBMSC sheet (e) and one single cell as a negative control (f) observed and imaged under SEM. H&E staining of rBMSC sheet (g). Scale bar: (a), (d): 200 *μ*m; (b), (c): 100 *μ*m; (g): 25 *μ*m.

In addition, the ability of reattachment also represented the viability of rBMSC sheets. The rBMSC sheets were harvested and subjected to reattachment analysis on traditional plates and titanium disks which were often used in dental implant research. The results showed that obtained rBMSC sheets could reattach well on 24-well plate (Figure 3(a)) and titanium disks (Figures 3(b) and 3(c)). Reattached rBMSCs migrated out of the sheets at the edges and a large number of cells comprised the sheets in the center. The display of collagen type I of cell sheets was shown in Figure 3(d). We found that cell sheets had quantities of collagen type I. Moreover, the morphology of detached cell sheets and cells on TiO_2_ nanodot films was observed by SEM. As shown in Figure 3(e), cell sheets had rich ECM, and cells were connected with each other in a dense network-like tissue. In the negative control group (Figure 3(f)), one single cell spread on TiO_2_ nanodot films with pseudopodia.

### 3.5. H&E Staining and Thickness of rBMSC Sheet

To study the structure of cell sheets, H&E staining assay was conducted. As shown in Figure 3(g), the cultured cell sheet was composed of 4 to 8 layers of cells at day 5. The thickness of rBMSC sheets was measured by laser confocal microscopy.

The rBMSC sheets grew thicker with culturing, from an initial 26.7 ± 1.5 *μ*m (day 5) up to 47.7 ± 3.0 *μ*m (day 10) (Figure 2(c)). All together, rBMSC sheets grew well on laminin-521-coated nanodot films.

### 3.6. Differentiation of rBMSC Sheet

**Figure 4:**
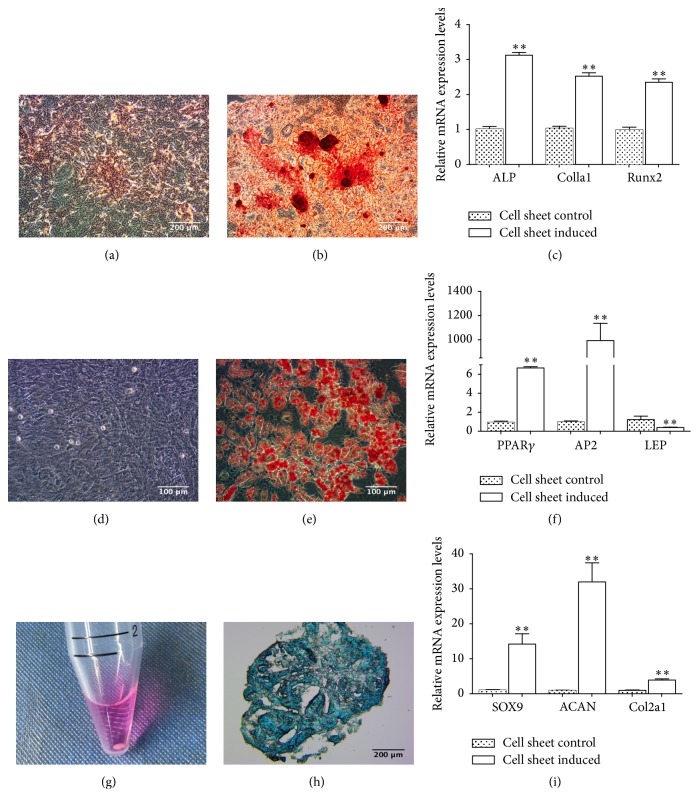
Multilineage potential of rBMSC sheets. Alizarin-red staining was performed after osteogenic differentiation of cell sheets for 21 days (b). The control group was cultured in cell sheet nutrient solution (a). Oil Red O staining was performed after adipogenic induction of cell sheets for 21 days (e). As the control group, cell sheets were cultured in cell sheet nutrient solution (d). A small pellet formed after chondrogenic induction for 28 days (g). The pellet was cut into slices and stained with Alcian Blue (h). Relative mRNA expression levels in the osteogenic (c), adipogenic (f), chondrogenic (i) induced cell sheet group compared with that of the negative control group. ^*∗∗*^*P* < 0.01. Scale bar: (a), (b), and (h): 200 *μ*m; (d), (e): 100 *μ*m.

In order to ensure multilineage potential of fabricated rBMSC sheets, osteogenic, adipogenic, and chondrogenic differentiation of these cell sheets were conducted. Cell sheets were cultured in osteogenesis-inducing liquid and subjected to alizarin-red staining. From inverted microscopy, red calcium-nodule formation was observed (Figure 4(b)), but there was no obvious calcium nodule in the control group (Figure 4(a)).  Figure 4(c) showed the expression levels of* ALP, col1a1*, and* runx2* in each group. The expression levels in the osteogenesis-induced cell sheet group were significantly higher than in the negative control group (*P* < 0.01).

With Oil Red O staining, rBMSC sheets had red lipid droplets after 3 weeks of adipogenic differentiation (Figure 4(e)), while there was no stain in the negative control group (Figure 4(d)). At the end of the first cycle of induction, lipid droplets were observed and the number and size of lipid droplets increased with culturing. At day 7, the mRNA expression levels of* PPARγ* and* AP2* were higher than the negative control group, while* leptin* was downregulated (Figure 4(f)) (*P* < 0.01).

After 28 days of chondrogenic induction, rBMSC sheets formed a visible cartilage sphere (Figure 4(g)). The tissue section was blue after staining with Alcian Blue (Figure 4(h)). This dye can stain glycosaminoglycans in cartilages. At day 7, the mRNA expression levels of* SOX9, ACAN*, and* col2a1* significantly increased compared with the negative control group (Figure 4(i)) (*P* < 0.01).

## 4. Discussion

In this study, a novel method to generate a multipotential sheets of rBMSCs on laminin-521-coated nanodot films has been developed. According to our results, coating of nanodot films with 1.2 *μ*g/mL laminin-521 can provide beneficial conditions for efficient attachment and growth of rBMSCs. The results of live-dead staining, proliferation assay, and reattachment assay indicate that the obtained rBMSC sheets have good viability and reattach well on plates and titanium disks. Our data suggests that human recombinant laminin-521 and UV365 are safe for rBMSC sheet harvesting. Moreover, rBMSC sheets maintain multilineage potential, including osteogenic, adipogenic, and chondrogenic differentiation.

In our study, rBMSC sheets were comprised of 4 to 8 layers of cells. This result was consistent with previous studies [15, 27]. The reason why rBMSCs piled up 4 to 8 layers was not investigated further. But it is estimated that this phenomenon is connected with the feature of BMSCs. A study found that human BMSCs could locally grew into multilayers, and contact inhibition of BMSCs was not apparent [28]. During the culture of rat BMSCs, we found the same phenomenon. In addition, the results of live and dead staining showed that only a few of cells were dead. Kikuchi et al. [29] found that there were thickness limitations in maintaining tissues in a static culture. Three-layer human skeletal muscle myoblast (HSMM) sheets maintained their live cell number. One-layer HSMM sheet was comprised of more than three-layer cells. Three-layer HSMM sheets could have more than nine-layer cells. So, it is possible that multilayered rBMSC sheets have a few of dead cells. In addition, in our study, in order to prevent the necrosis of cell sheet, the culture medium was replaced every day when cells were confluent. The results of H&E staining and SEM showed that there were some spaces among cells. It is estimated that oxygen and nutrients can be exchanged through these spaces.

In previous studies, rBMSC sheets were obtained by mechanical scraping [5, 15] and temperature-controlled methods [27]. However, mechanical scraping can cause loss of cells and damage to cell sheets. The temperature-controlled methods provided a way to harvest rBMSC sheets. On a thermally responsive poly(N-isopropylacrylamide)-coated culture surface, confluent BMSCs were incubated at 20°C for 30 min and then rBMSC sheets were detached spontaneously and allowed to float up into the medium. But it was complex and needed long time to prepare poly(N-isopropylacrylamide)-coated surfaces [30]. As an alternative, light-induced methods have been developed in this study to obtain rBMSC sheets. On the laminin-521-coated films, dispersed rBMSCs attached and proliferated into intact contiguous cell sheets, which were then harvested by simply illuminating the films with UV365. The results of this study indicated that UV illumination was safe for cell sheet harvesting, which was similar to low temperature.

In our study, it was prerequisite to precoat the nanodot films with laminin-521 to promote rBMSCs adhesion. Laminin-521 significantly promotes BMSCs adhesion, which is in agreement with a previous study [23]. Rapid adhesion on laminin-521-coated nanodot films seems to contribute to rBMSC sheet formation. Although laminin-521 is confirmed to promote adhesion, the adequate mechanism remains unclear. It has been reported that E-cadherin and integrin *β*1 may play some important roles in the rapid adhesion on laminin-521 [22]. E-cadherin, which is known as the primary cell-to-cell adhesion molecule, was shown to enhance hiPSCs cell proliferation on laminin-521 [31]. When integrin *β*1 was blocked by functional antibodies, adhesion of cells were significantly reduced. Moreover, another report [23] provided evidence that CD49f as a stemness marker of BMSCs was also correlated with cell adhesion on laminin-521. Thus, one possible explanation is that enhancement of rBMSCs adhesion by laminin-521 maintains the proliferative potential, which in turn enables a higher saturation density, when compared to cells cultured on uncoated control in this study.

In addition, a previous study confirmed that the activation of PI3K/Akt pathway could inhibit anoikis in stem cells on laminin-521, which was necessary for cell survival [22]. Furthermore, Laperle et al. [32] proposed that *α*-5 laminin self-renewal signaling combined with other soluble signals could drive stem cells to produce a matrix in an autocrine and paracrine signaling loop. Disrupted *α*-5 laminin production caused apoptosis and impaired self-renewal, while cells cultured on laminin-521 rescued this phenomenon. Moreover, cells on laminin-521 had a lower tendency to aggregate, since laminin-521 did not have *α*-1 and *β*-1 arms [33]. Without local aggregation, rBMSCs formed cell sheets successfully. Therefore, the coated laminin-521 during in vitro culture of rBMSC sheets is considered to be beneficial for cell culturing.

Furthermore, in order to ensure differentiation capability of rBMSC sheets, osteogenic, adipogenic, and chondrogenic differentiation were investigated. The results were in agreement with a previous study [34] and implied that hyperconfluent rBMSC sheets still had capability of multilineage differentiation. However, multipotency maintenance mechanism of rBMSC sheets was unclear. It can be explained that differentiation of BMSCs into osteogenic, adipogenic, and chondrogenic lineages required a specific cocktail of supplements to be added to the basal medium. In addition, a study found that cells cultured on laminin-521 did not increase the DNA damage when grown at high density [35]. Thus, laminin-521 seems to provide beneficial condition for cell culturing.

Moreover, laminin-521 was reported as capable of stabilizing the pluripotent phenotype of pluripotent human embryonic stem cells* via α*6*β*1 integrin signaling [20]. The expression levels of the pluripotency markers Oct4, Nanog, and Sox2 were more stable on laminin-521-coated substrates than the control group [22]. However, understanding of the effects of laminin-521 on rat derived BMSC sheet construction is limited, as there are no studies investigating the effects of laminin-521 on rBMSCs. In above cited studies, cells are all human derived. Future studies will aim to uncover the mechanism of promoting rat derived BMSCs adhesion on laminin-521-coated nanodot films and determine whether rBMSC sheets are indeed capable of providing the necessary framework for cell sheet-based therapies in vivo.

In this study, we successfully constructed rBMSC sheets using laminin-521 through light-induced cell sheet technology. This method provides a simple, rapid, and effective strategy to harvest rBMSC sheets. Obtained rBMSC sheets preserve multilineage potential which lays the foundation for various cell sheet-based therapies.

## 5. Conclusion

In summary, laminin-521 and UV365 illumination systems provided a simple, rapid, and effective cell sheets strategy. The fabricated rBMSC sheets had good viability using light-induced cell sheet technology. Moreover, the hyperconfluent rBMSC sheets were still capable of multilineage potential.

## Figures and Tables

**Table 1:** Nucleotide sequences for real-time RT-PCR primers.

Genes	Sequences of primer (5′–3′)
*ALP*	Forward: TGGTACTCGGACAATGAGATGC
	Reverse: GCTCTTCCAAATGCTGATGAGGT
*Col1a1*	Forward: AGAGGCATAAAGGGTCATCGTG
	Reverse: CAGGTTGCAGCCTTGGTTAGG
*Runx2*	Forward: CAGTATGAGAGTAGGTGTCCCGC
	Reverse: AAGAGGGGTAAGACTGGTCATAGG
*PPARγ*	Forward: CCCTTTACCACGGTTGATTTC
	Reverse: CTTCAATCGGATGGTTCTTCG
*AP2*	Forward: CTTGGTCGTCATCCGGTCAG
	Reverse: CCAGGGTTATGATGCTCTTCACT
*LEP*	Forward: CTTTGGTCCTATCTGTCCTATGTTC
	Reverse: GAGGATCTGTTGATAGACTGCCA
*SOX9*	Forward: AGGCCACCGAACAGACTCAC
	Reverse: GAAGGTCTCGATGTTGGAGATGA
*ACAN*	Forward: ACAGACACCCCTACCCTTGCT
	Reverse: CCTCACATTGCTCCTGGTCG
*Col2a1*	Forward: GTGGAAGAGCGGAGACTACTGG
	Reverse: TTGGGGTAGACGCAAGACTCG
*β-Actin*	Forward: TGCTATGTTGCCCTAGACTTCG
	Reverse: GTTGGCATAGAGGTCTTTACGG
